# Self-Reported Long-Term Antiretroviral Adherence: A Longitudinal Study Among HIV Infected Pregnant Women in Mpumalanga, South Africa

**DOI:** 10.1007/s10461-019-02563-z

**Published:** 2019-06-21

**Authors:** Shandir Ramlagan, Violeta J. Rodriguez, Karl Peltzer, Robert A. C. Ruiter, Deborah L. Jones, Sibusiso Sifunda

**Affiliations:** 1 Social Aspects of Public Health, Human Sciences Research Council, Private Bag X41, Pretoria, 0001 South Africa; 2 Department of Work & Social Psychology, Maastricht University, P.O. Box 616, 6200 MD Maastricht, The Netherlands; 3 Department of Psychiatry and Behavioral Sciences, Miller School of Medicine, University of Miami, 1400 NW 10th Ave, Miami, FL 33136 USA; 4 Department of Psychology, University of Georgia, Athens, GA 30605 USA; 5 Department of Research and Innovation, University of Limpopo, Sovenga, 0727 South Africa

**Keywords:** ART adherence, Maternal, Pregnancy, South Africa, Randomized Control Trial

## Abstract

We evaluate the impact of a multi-session cognitive behavioral prevention of mother to child transmission (PMTCT) intervention on antiretroviral therapy (ART) adherence. A total of 683 women were enrolled into a randomized control trial conducted at twelve community health centres (CHCs) in Mpumalanga Province. Participants were randomized to Standard Care or Enhanced PMTCT Intervention (EI). EI received three group and three individual intervention sessions. EI impact was ascertained on ART adherence (baseline vs 12 months post-partum). Women in the intervention groups were less likely to remain stable with regards to ART adherence over time compared to the control groups. In predicting if women become adherent over time, the intervention condition had no impact. However, the intervention condition was significantly positively associated with change to non-adherence. The enhanced cognitive-behavioral PMTCT intervention did not show any improvement in relation to maternal ART adherence relative to standard PMTCT care.

**Trial registration** Clinicaltrials.gov: number NCT02085356.

## Resumen

Evaluamos el impacto de una intervención cognitiva de comportamiento para la prevención de la transmisión maternoinfantil (PTMI) a través de la adherencia a la terapia antirretroviral (TAR). Un total de 683 mujeres se inscribieron en un ensayo aleatorio de control realizado en doce centros de salud comunitarios (CHC) en la provincia de Mpumalanga. Los participantes fueron asignados al azar a Atención estándar o Intervención PMTCT mejorada (EI). La condición IE recibió tres sesiones grupales y tres individuales. El impacto de la IE se determinó en la adherencia al tratamiento antirretroviral (primera sesión a 12 meses después del parto). Las mujeres en los grupos de intervención tuvieron menos probabilidades de permanecer estables con respecto a la adherencia al tratamiento antirretroviral en comparación al grupo de control. Al predecir si las mujeres comenzaron a adherirse a su tratamiento antiretroviral 12 meses después, la condición de intervención no tuvo efecto. Sin embargo, la condición de intervención se asoció significativamente de manera positiva al lograr que las mujeres no pararan de tomar su tratamiento antiretroviral 12 meses después. La intervención de PTMI cognitiva-conductual mejorada no mostró mejoría en relación a la adherencia al TAR materno en comparación con la atención de PTMI estándar.

## Introduction

Women who are pregnant and living with Human Immunodeficiency Virus (HIV) are recognised as part of the HIV key and vulnerable population group [1, 2]. During pregnancy, antiretroviral therapy (ART) is recommended for HIV infected women for viral suppression and reduction of perinatal HIV transmission [3, 4]. It is not simply the provision of the ART that is recommended but also the adherence regimen associated with ART. In this case, adherence to ART is defined as patients taking their medication as prescribed, as even minor deviations from this regimen can be detrimental to maternal and neonatal health [5].

The importance of adherence to ART for HIV has been documented in research literature [4–8]. Studies have found that ART adherence lower than 95% can be associated with the development of viral resistance to medications whereas adherence above 95% is associated with no opportunistic infection nor deaths [9, 10]. Reasons for ART non-adherence include side-effects, being away from home, lack of food and medication, non-disclosure of HIV status to partner, stigma, and work-related demand [11]. Interestingly, longitudinal studies in Argentina, Brazil, Peru [4] and in Switzerland [5] found that self-reported adherence among HIV infected pregnant women decreased significantly post-partum.

Similar findings are reported by Nachega et al. [12] in his meta-analysis of 51 articles of pre- and post-natal HIV infected women from across the world, including seven from South Africa. It was found that overall, that is during and after pregnancy, an estimated 74% of pregnant women were adequately (> 80%) adherent to their ART regime. Adherence was much higher during pregnancy then after pregnancy (76% vs 53% respectively) [12]. The decrease in ART adherence during the post-partum period was clarified as the mother’s concern regarding the transmission of her HIV to her fetus during pregnancy and birth had abated [12]. This is explained by the necessity concerns framework which postulates that a person will adhere to the medication regime if they see a necessity to take the medication and have no concerns about its adverse effects [13, 14]. If no necessity is experienced anymore and a concern about adverse effects is seen, medication adherence will decrease.

As of 2016, South Africa had the biggest HIV epidemic in the world with 7.1 million people living with HIV, 270,000 new infections annually, 110,000 AIDS related deaths, and the largest ART program in the world with 56% of adults on ART [15]. In terms of HIV infected pregnant women, 95% received ART in 2016 and mother-to-child transmission (MTCT) of HIV fell from 3.6% to 1.5% between 2011 and 2016 [16]. As such, it is possible to eliminate MTCT in South Africa. Peltzer et al. [17] noted that successful PMTCT interventions in South Africa have included mother-to-mother peer mentoring as well as cognitive behavioral interventions [18]. Male involvement in PMTCT was also seen as improving PMTCT outcomes [19] as well as interventions that involved numerous text messages and telephone calls to pregnant women reminding them of PMTCT [20]. In the 51 study meta-analysis of ART adherence during and after pregnancy, facilitators of better adherence included higher education, higher income, knowledge about PMTCT, previous PMTCT, disclosure of HIV status, positive partner support, support groups, and being on lifelong ART [12]. Barriers to ART adherence include but not limited to being younger, drug use, depression, home births, and number of pills [12].

Little is known about longitudinal ART adherence among HIV infected pregnant women in South Africa. The present study aimed to longitudinally examine the impact of a prevention of mother to child transmission (PMTCT) uptake intervention on ART adherence among HIV infected pregnant women in Mpumalanga province, South Africa. It was hypothesised that in the experimental condition mothers would be significantly more likely to adhere to ART protocol medications as prescribed compared to control condition mothers when comparing pre-natal and post-partum adherence rates.

## Methods

### Study Design

**Fig. 1:**
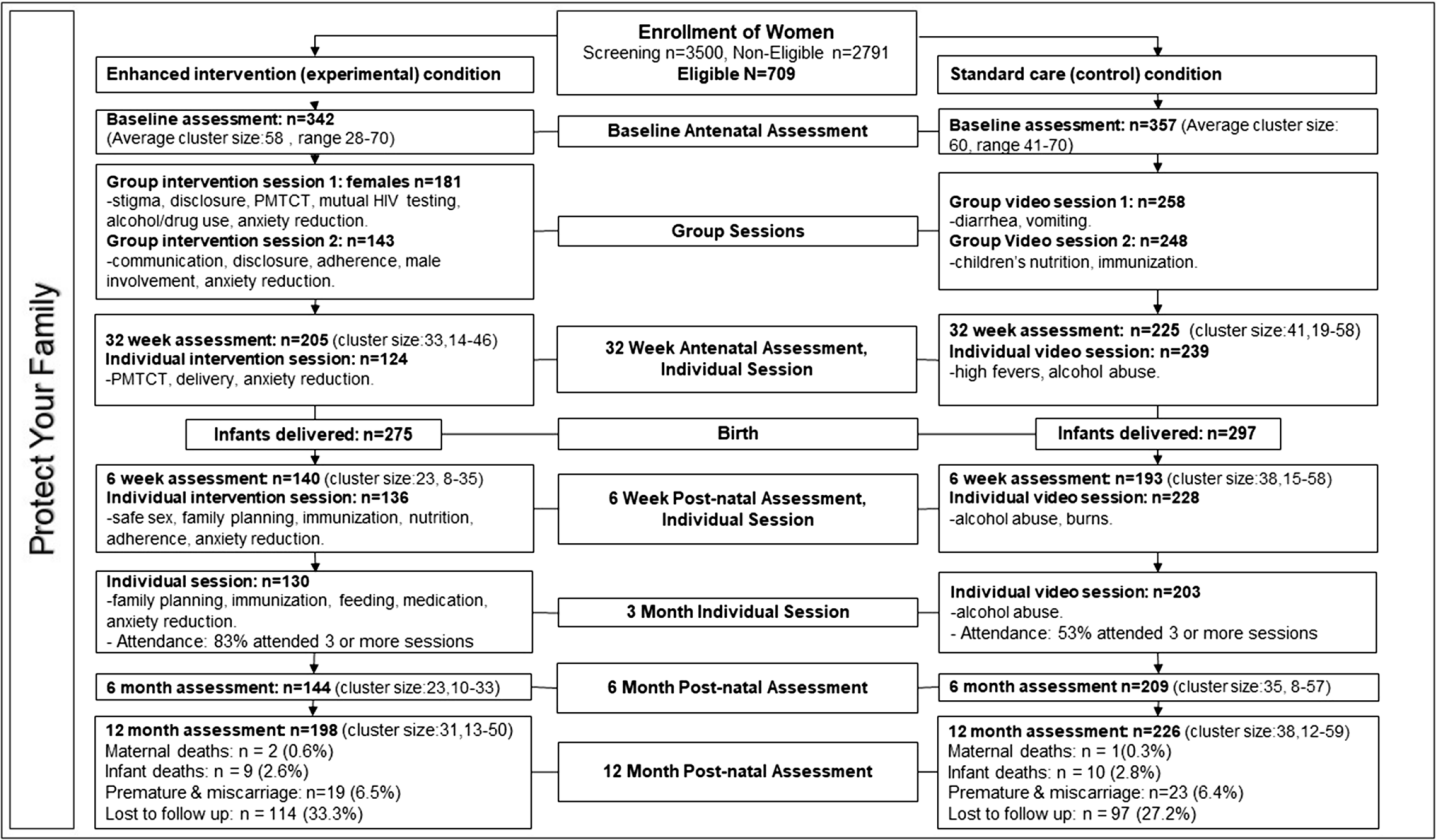
“Protect Your Family” RCT design

Study data were drawn from the ‘Protect Your Family’ (PYF), longitudinal, clinic-randomized, PMTCT controlled trial, conducting a baseline assessment prenatally, and a long-term follow up assessment post-partum [21]. The baseline assessment was conducted when the women were between 8 and 24 weeks pregnant (M = 18 weeks, SD = 5.47) while the long-term follow up post-partum assessment was conducted 12 months after birth (see Fig. 1). Although the cluster randomized controlled trial (RCT) gathered intermediate measures at 32 weeks pre-natal, 6 weeks and 6 months post-natal, the loss to follow-up was too high to warrant analyses of that data for this paper (Fig. 1). The RCT was conducted in 12 community health centres (CHCs) in Gert Sibande and Nkangala districts in Mpumalanga province, South Africa. Randomization was done where HIV infected pregnant women either received a Standard Care (SC) condition or an Enhanced Intervention (EI) condition (Fig. 1) [17, 21].

### Interventions

#### Enhanced Intervention (Experimental) Condition (EI)

EI participants received the standard PMTCT care offered at all South African CHCs as well as the two group and three individual theory-based social-cognitive PYF intervention (see Fig. 1). The standard care consisted of CHC administered Option B treatment, where the test and treat model was utilized, ensuring that as soon as the pregnant woman was diagnosed as having HIV, she would immediately be enrolled onto ART which would continue after birth only if the mothers health required it [22–24]. The two PYF group intervention sessions of approximately 2 h each were held between baseline (8–24 weeks pregnant) and 32 weeks pregnancy [17, 21]. The groups consisted of between five to seven participants and sessions were conducted in a private room at the CHC the women were recruited. The facilitators of each session were trained PYF staff member who were lay councillor and were independent of the CHC. Three individual PYF intervention sessions occurred just prior to birth (32 weeks pregnant), as well as 6-weeks and 3 months post-partum.

The PYF EI is a manualized, closed, structured behavioural risk reduction program targeting PMTCT, partner violence, stigma, disclosure, alcohol/drug use, anxiety reduction, communication, disclosure, ART adherence, male involvement, family planning, delivery, safe sex, immunization, nutrition, feeding, and medication (Fig. 1) [21]. During the intervention, participants received cognitive behavioural skills training addressing the key components of each session. Participants were encouraged by the PYF staff member to problem solve, provide supportive feedback to each other, and peer mentor one another [21]. Individual PYF sessions included one-on-one counselling and information sharing sessions where participants were provided with discrete intervention. Thus the intervention as a whole sought to change behaviour as well as influences on behaviour both within the individual and externally by not only providing valuable in-depth information but also by allowing participants to experience the views of others in a similar situation as theirs and gain from the collective. In terms of ART adherence, participants underwent training on what PMTCT is and what it means for them individually and as a group, ART pre- and post-natal, ART for baby, ways to prevent babies from being infected with HIV, the importance of adherence to medication, how to adhere to medication, concerns about ART, confusion and side effects surrounding ART, skipping or missing ART, as well as problems with taking ART including lack of food, transport to get to the CHC, etc. Details regarding the full PYF EI can be found in Jones et al. [21].

#### Standard Care (Control) Condition (SC)

SC participants received the standard PMTCT care (25) offered at all South African CHCs as well as health videos on diarrhoea, vomiting, children’s nutrition, immunization, high fevers, alcohol abuse and burns [21]. The standard PMTCT care offered by all CHCs included immediate ART initiation should the pregnant women not be on treatment as part of antenatal care, labour and delivery care, as well as postnatal care including baby health, feeding and development [25]. In addition to the standard PMTCT care offered by CHC nurses, women enrolled into the SC condition were shown general health videos utilising a 40 inch flat screen LCD television paired with a DVD player in a private room at the CHC the women were recruited. This ensured that SC participants would spend the same amount of time at the CHC as the EI condition participants. The SC condition also followed the same protocol timing as the EI condition (see Fig. 1).

### Sample and Procedure

Women were recruited from the 12 sample CHCs while attending ante-natal care. CHCs were matched according to patient turnover and average antenatal care (ANC) volume which produced 6 matched pairs. In each of the 1:1 matched pair, a computer program was utilized to randomly assign each CHC as either EI or SC [21]. This cluster randomization allowed for all participants in a specific CHC to receive the exact same condition and thus cross study contamination could be contained. ANC nurses at each of these 12 CHCs, utilizing the indicators of HIV positive and less than 6 months pregnant, referred potential participants to the PYF staff based at the CHC [24]. The PYF staff then utilized the study inclusion criteria of being an HIV infected pregnant women, being less than 6 months pregnant, aged 18 years and older, having a current partner and not been recruited in this study at another CHC to consecutively sample participants [21, 24]. Once screened, eligible women were provided with an informed consent form in a language of their preference including English, isiZulu, or seSotho. The information sheet and consent form were read to women who followed along using their own copies. Those women who agreed to enter the survey were required to provide written consent. Recruitment for this RCT began in April 2014 and ended in April 2015. The trial concluded in March 2017 when the last participant completed a 12 month post-partum assessment.

Once consented, women completed a baseline assessment utilizing the Audio Computer-Assisted Self-Interview (ACASI) technology [26] loaded onto a touch screen Lenovo ThinkPad X230 tablet/laptop (with 180° swivel screen) and connected with a Logitech h150 stereo over-ear headphones set [24]. Trained fieldworkers provided all women with basic training on the usage of the ACASI software as well as the hardware. Training on ACASI was done utilizing the initial demographic variables of the assessment. Once women were comfortable with ACASI, the fieldworkers moved aside to provide confidentiality but stayed in the room should the women have required them. All women who completed a baseline assessment was provided R50.00 (South African Rand = US$4.72) and R100 for 12 month post-partum assessment.

### Measures

#### Antiretroviral Adherence

The main outcome measure of this study was maternal adherence to ART. In South African, during the study time period, pregnant women attending ante-natal clinics were offered free Option B treatment including tenofovir (TDF) + emtricitabine (FTC) or lamivudine (3TC) + efavirenz (EFV) as fixed dose combinations [25, 27]. Adherence to these medications were assessed by the Adult AIDS Clinical Trials Group (AACTG) [28] self-reported antiretroviral (ARV) adherence measure. The assessment included a 4 day recall on the number of ARV medication doses missed that is (a) yesterday (b) the day before yesterday, (c) 3 days ago and (d) 4 days ago. Participants’ responses were dichotomized into a score of 0 to indicate that they missed medication in the past 4 days (non-adherent) or 1 if no medication was missed in the past 4 days (adherent).

#### Reasons for Missing ART

The AACTG was utilised to understand the reasons for non-adherence [28]. The instrument includes 14 possible reasons for missing ART. Responses to each reason were dichotomized to 1 (Yes) and 0 (No).

#### Sociodemographic, Alcohol, Reproductive, HIV, and Partner Information

At baseline, personal information about the participants were collected regarding age, educational attainment, relationship status, employment status, alcohol use, income, number of children, if this pregnancy was unplanned, if the women had any HIV infected children, if the women were diagnosed with HIV during this current pregnancy, time in months since ART initiation, if partner is HIV infected, and disclosure of HIV status to partner.

#### Male Involvement

An 11-item “Male Involvement Index” assessed the women’s partner’s involvement during the antenatal period [21]. Questions included “Does your male partner attend antenatal care visits with you,” “Have you discussed antenatal HIV prevention for your baby with your male partner,” etc. Participants responded to each item as 1 (Yes) or 0 (No), and scores ranged from 0 to 11. Cronbach’s α reliability coefficient for the scale at baseline was α = 0.83, and at 12 month post-partum α = 0.82.

#### Intimate Partner Violence (IPV)

The Conflict Tactics Scale 18 [29, 30] was used to assess psychological victimization (9-item) and physical violence (9-item) in the past month. The 18 conflict situations presented to women included “discussed the issue calmly,” “threw something at you,” etc. in the previous month. Responses were recorded on a seven point scale of 0 (never) to 6 (more than 20 times) where a higher score is indicative of increased IPV. Cronbach’s Alpha reliability for the psychological victimization subscale at baseline was α = 0.76, and at 12 month post-partum α = 0.83. Cronbach’s α reliability coefficient for physical violence subscale at baseline was α = 0.92, and at 12 month post-partum α = 0.94.

#### Stigma

The nine-item AIDS-Related Stigma Scale [31] measured externalized stigma as experienced by the women. Items in the scale included “People who have AIDS should be ashamed,” “People who have HIV should be isolated,” etc. Response options were dichotomous to 0 (disagree) and 1 (agree) resulting in a total scores range of 0 to 9, where higher scores indicate greater levels of stigma. Due to poor internal reliability (α = 0.58), the reversed coded item for this scale, question 4, was excluded. Excluding the item, Cronbach’s α reliability coefficient for the scale improved to α = 0.74 at baseline, and α = 0.69 at 12-months.

#### Depression

The 10-item “Edinburgh Postnatal Depression Scale 10” [32] measured the severity of depression experienced by the women. Women were asked to rate how often they had experienced different symptoms associated with depression during the previous 7 days. Questions included “I have been able to laugh and see the funny side of things,” “I have felt sad or miserable,” etc. Scores ranged from 0 through 30 where the higher the score, the more the likelihood of depression being experienced. Validated cut-off score for South African populations is 12 [33]. Cronbach’s α reliability coefficient for the scale at baseline was α = 0.66, and at 12 month post-partum α = 0.67.

#### Data Analysis

Means, standard deviations, frequencies, and percentages were used to describe the sample. T-tests and Mann-Whiney tests, depending on distributional assumptions, as well as Chi square tests were used to compare adherent and non-adherent participants. Multinomial logistic regression was used by comparing prenatal adherence at baseline with 12 months post-partum adherence. The dependent variable consisted of women reporting nonadherence prenatally and at 12 months follow-up (reference category), women reporting being adherent at baseline and 12-months follow-up, women who began reporting adherence from baseline to 12 month follow-up, and women who stopped reporting being at adherent at 12 months follow-up from baseline. Odds ratios with 95% confidence intervals were calculated as effect sizes for each of the outcomes [34]. Missing data was handled using multiple imputation with ten imputed datasets [35]. The multinomial logistic regression analyses were conducted using Mplus version 7.4 [36] and all other analyses were conducted using SPSS V24.

## Results

### Attrition Analysis

A total of 683 HIV infected pregnant women completed a baseline assessment and 403 completed a 12 month post-partum assessment. In multivariable logistic regression, education (AOR = 1.68, p = 0.014], decreased depression (AOR = 0.69, p = 0.045], and antiretroviral adherence (AOR = 1.47, *p* = 0.045] were associated with retention in PMTCT care (all *p*s < 0.05), after controlling for HIV-related stigma and infant HIV status. No other variables were associated with attrition. Variables associated with attrition were included as covariates if they were associated with the outcomes.

### Baseline Differences Between Conditions

Age, education, relationship status, monthly income, number of children, depressive symptoms, and adherence were not different between conditions at baseline. However, the partners of women in the control condition were marginally more likely to be HIV infected (28.4% versus 21.7%, *p* = 0.052). Variables that were statistically different between conditions at baseline were included as covariates if they were associated with the outcomes.

### Sample Characteristics at Baseline

The mean age of the women was 28.40 (SD = 5.71) years (see Table 1), with a range of 18 to 46 years. A total of 194 women or 29% had completed school, 83% were not employed, 45% earned less that R600 per month, and 59% were not married and living separately. Just over half (53%) of women reported that this current pregnancy was unplanned and a fifth (20%) reported that they had no children. Of those that have children, 5% reported to know that their child is HIV infected. Over half (54%) of the women were diagnosed with HIV during this pregnancy with the majority (60%) having disclosed their HIV status to their partner. The mean time since ART initiation among women was 13.27 (SD = 24.35) months. Over half (54%) of women reported male involvement during this pregnancy and 13% reported that they had drunk three or more alcoholic beverages on at least one occasion in the past month.

**Table 1:** ART adherence by socioeconomic, reproductive, HIV, partner, alcohol use, stigma and depression at baseline (N = 683)

	All (*N *= 683)	Nonadherent (N = 224)	Adherent (N = 457)	*Z/t/χ* ^*2*^ *,p*
	Mean (SD)	Mean (SD)	Mean (SD)	
	n (%)	n (%)	n (%)	
Sociodemographics				
Age (m, SD)	28.40 (5.71)	28.87 (5.77)	28.17 (5.67)	1.514, 0.131
Educational attainment				
Grade 0–9	148 (21.7%)	67 (29.9%)	81 (17.7%)	**14.998, 0.001**
Grade 10–11	339 (49.8%)	107 (47.8%)	232 (50.8%)	
Grade 12 or more	194 (28.5%)	50 (22.3%)	144 (31.5%)	
Relationship status				
Not married, living separate	403 (59.2%)	129 (57.6%)	274 (60.0%)	0.382, 0.826
Not married, living together	153 (22.5%)	53 (23.7%)	100 (21.9%)	
Married	125 (18.4%)	42 (18.8%)	83 (18.2%)	
Employment status				
Not employed	562 (82.5%)	185 (82.6%)	377 (82.5%)	0.001, 0.976
Employed, Volunteer or Student	119 (17.5%)	39 (17.4%)	80 (17.5%)	
Income (ZAR) per month				
< 600	308 (45.2%)	97 (43.3%)	211 (46.2%)	0.499, 0.480
≥ 600 or more	373 (54.8%)	127 (56.7%)	246 (53.8%)	
Reproductive				
Number of children				
None	139 (20.4%)	35 (15.6%)	104 (22.8%)	**4.707, 0.030**
One or more	542 (79.6%)	189 (84.4%)	353 (77.2%)	
Unplanned pregnancy				
No	320 (47.0%)	95 (42.5%)	225 (49.2%)	2.810, 0.094
Yes	361 (53.0%)	129 (57.6%)	232 (50.8%)	
HIV issues				
Diagnosed with HIV in this pregnancy				
No, before	314 (46.1%)	120 (53.6%)	194 (42.5%)	**7.481, 0.006**
Yes	367 (53.9%)	104 (46.4%)	263 (57.5%)	
Months since ART initiation	13.27 (24.35)	16.41 (27.09)	11.73 (22.76)	− 4.146, < 0.001*
Partner issues				
Disclosed HIV status to partner				
No	279 (41.0%)	71 (31.7%)	208 (45.5%)	**11.867, 0.001**
Yes	402 (59.9%)	153 (68.3%)	249 (54.5%)	
Male involvement (cut of ≥ 8 (median score of male involvement))				
No	313 (46.0%)	102 (45.5%)	211 (46.2%)	0.024, 0.876
Yes	368 (54.0%)	122 (54.5%)	246 (53.8%)	
Intimate partner violence				
Psychological partner violence	3.24 (5.31)	3.86 (5.63)	2.94 (5.14)	**− 4.718, 0.007**
Physical partner violence	1.14 (3.68)	2.07 (5.15)	0.69 (2.57)	**–3.498, < 0.001**
Alcohol use, stigma and depression				
Alcohol use of more than 2 drinks on at least on one occasion in the past 4 weeks				
No	587 (86.2%)	178 (79.5%)	409 (89.5%)	**12.716, < 0.001**
Yes	94 (13.8%)	46 (20.5%)	48 (10.5%)	
Stigma	0.773 (1.36)	0.69 (1.28)	0.97 (1.49)	− 2.541, 0.011*
Depression				
EDS score of 0–12	349 (51.2%)	87 (38.8%)	262 (57.3%)	**20.572, < 0.001**
EDS score of 13 and more	332 (48.8%)	137 (61.2%)	195 (42.7%)	
Intervention				
Standard of care	345 (50.7%)	100 (44.6%)	245 (54.6%)	**4.836, 0.028**
Enhanced intervention	336 (49.3%)	124 (55.4%)	212 (46.4%)	

*Mann–Whitney tests were used for median comparison of groups and Chi square tests for differences in proportions

In Table 1, ART adherence at baseline assessment was positively associated with higher educational attainment, number of children, disclosure of HIV status to partner, and low reported alcohol use. The findings show that physical partner violence, time since ART initiation in months, psychological partner violence, and stigma were negatively associated with adherence to ART.

### ART Adherence Change

Table 2 below presents the multinomial logistic regression analyses, which shows the odds of women with sustained adherence, changing to adherent and changing to non-adherent from baseline (8–24 weeks prenatal) to 12 months post-partum. In predicting sustained adherence, a significant effect of condition was found suggesting that women in the enhanced intervention condition were less likely to sustain ART adherence over time than women in the standard care condition, after controlling for alcohol use, intimate partner violence, and depressive symptoms. In predicting if women become adherent over time, the intervention condition had no impact on women becoming adherent when controlling for other factors. The intervention condition was significantly associated with change to non-adherence from baseline to 12-month follow-up, which may indicate that the intervention had no long term effect on remaining adherent.

**Table 2:** Multinomial logistic regressions with “Stable non-adherence” (prenatal and 12 months postnatal) as reference group (n = 39)

	Sustained adherence (n = 220)		Change to adherent (n = 90)		Change to Non-adherent (n = 54)	
	OR [95% CI]	AOR [95% CI]	OR [95% CI]	AOR [95% CI]	OR [95% CI]	AOR [95% CI]
*Fixed effects*						
Intervention	0.528 [0.355, 0.785]**	0.601 [0.396, 0.911]*	1.196 [0.748, 1.912]	1.021 [0.629, 1.658]	2.474 [1.353, 4.526]**	2.182 [1.177, 4.050]*
Covariates (baseline)						
Age	0.982 [0.948, 1.018]	**–**	1.018 [0.976, 1.062]	**–**	1.013 [0.962, 1.067]	–
Educational attainment (ref = up to 10 years)						
10 to 11 years	1.489 [0.892, 2.484]	1.452 [0.847, 2.487]	0.637 [0.355, 1.142]	–	1.047 [0.507, 2.158]	–
12 years or more	2.186 [1.234, 3.872]**	1.810 [0.995, 2.487]	0.679 [0.355, 1.298]	–	0.686 [0.292, 1.612]	
Monthly income	1.386 [0.928, 2.071]	–	0.915 [0.568, 1.475]	–	0.608 [0.342, 1.082]^^^	0.709 [0.385, 1.307]
Relationship status (ref = unmarried living separate)						
Unmarried, living together	1.153 [0.700, 1.899]	–	1.389 [0.773, 2.495]	–	0.412 [0.168, 1.012]^^^	0.508 [0.203, 1.271]
Married	1.054 [0.620, 1.790]	–	1.750 [0.961, 3.186]	–	0.778 [0.357, 1.694]	0.922 [0.411, 2.070]
Pregnancy Unplanned	0.743 [0.501, 1.102]	–	1.694 1.046, 2.746]*	1.625 [0.993, 2.658]^^^	0.856 [0.483, 1.520]	–
Diagnosed during this pregnancy	1.358 [0.917, 2.013]	–	0.770 [0.481, 1.232]	–	1.059 [0.597, 1.879]	–
Months since ART initiation	1.001 [0.993, 1.009]	–	1.002 [0.993, 1.011]	**–**	0.990 [0.975, 1.004]	–
Alcohol use	0.364 [0.199, 0.666]**	0.440 [0.236, 0.820]*	1.924 [1.032, 3.584]*	1.829 [0.969, 3.352]^^^	2.382 [1.178, 4.815]*	1.950 [0.932, 4.078]^^^
Stigma	0.938 [0.805, 1.093]	–	1.014 [0.847, 1.215]		0.894 [0.692, 1.153]	–
Disclosure of HIV status to partner	0.760 [0.508, 1.136]	–	1.140 [0.704, 1.1845]	**–**	0.750 [0.421, 1.334]	–
Male involvement	0.997 [0.936, 1.063]	–	0.955 [0.886, 1.029]	**–**	1.001 [0.912, 1.099]	–
Psychological intimate partner violence	0.965 [0.930, 1.002]^^^	1.011 [0.960, 1.065]	0.991 [0.947, 1.037]	–	1.050 [1.004, 1.098]*	1.043 [0.995, 1.094]^^^
Physical intimate partner violence	0.873 [0.797, 1.956]**	0.886 [0.794, 0.988]*	1.001 [0.932, 1.075]	–	1.054 [0.985, 1.128]	–
Depression	0.521 [0.350, 0.776]**	0.604 [0.397, 0.21]*	2.155 [1.334, 3.481]**	2.031 [1.245, 3.311]**	1.084 [0.611, 1.925]	–
*Model fit*						
− 2LL (deviance)	− 374.101		− 314.773		− 375.482	
Number of parameters	9		6		8	
AIC/BIC	764.20/793.73		645.55/673.69		760.96/779.42	

AOR, adjusted odds ratio

Random effects indicates the estimated variances from random effects logistic regression model

***p < 0.001, **p < 0.01, *p < 0.05, ^^^p < 0.10

Sustained adherence was also associated with decreased alcohol use (AOR 0.440; 95% CI 0.236, 0.820), decreased depressive symptoms (AOR 0.604; 95% CI 0.397, 0.21), and decreased physical intimate partner violence (AOR 0.886; 95% 0.794, 0.988). Change to adherent was associated with increased depressive symptoms (AOR 2.031; 95% CI 1.245, 3.311) and change to non-adherent was not associated with any covariates at 95% or better.

### Reason for Missing ART

Table 3 below presents the reasons, by those who were non-adherent, for missing ART by time and condition. Overall, when comparing baseline to 12 month post-partum, the proportion of those giving varying reasons for non-adherence decreased for the majority of reasons. In the control condition, however, increases from baseline to 12 months post-partum were observed for reasons such as “simply forgot”, “had too many pills to take”, and “felt sick or ill.” In the enhanced intervention condition, increases from baseline to 12 months post-partum was observed for reasons such as “had problems taking pills at specified times (with meals, on an empty stomach, etc.)”, “had problems taking medication due to lack of food” and “ran out of pills.”

**Table 3:** Reason for missing ART by time point and condition

	Control		Enhanced intervention	
	Baseline (n = 106)	12 month post-partum (n = 45)	Baseline (n = 134)	12 month post-partum (n = 69)
Reasons	%	%	%	%
Were away from home	49.1	31.1	57.5	50.7
Were busy with other things	46.2	44.4	52.2	33.3
Had a change in routine	37.7	35.6	38.1	31.9
Simply forgot	**34.0**	**42.2**	44.8	30.4
Did not want others to notice you taking medication	42.5	28.9	40.4	29.0
Had problems taking pills at specified times (with meals, on an empty stomach, etc.)	39.6	20.0	**34.3**	**36.2**
Fell asleep or slept through dose time	33.0	31.1	38.1	33.3
Felt depressed or overwhelmed	35.8	35.6	43.3	26.1
Felt like the drug was toxic or harmful	39.6	20.0	35.8	34.8
Wanted to avoid the side effects	32.1	26.7	43.3	23.2
Had too many pills to take	**30.2**	**37.8**	40.3	23.2
Had problems taking medication due to lack of food	27.4	17.8	**32.8**	**33.3**
Felt sick or ill	**26.4**	**33.3**	30.6	23.2
Ran out of pills	16.0	13.3	**24.6**	**29.0**

## Discussion

The study examined the impact, over time, of a multi-session cognitive behavioral PMTCT intervention, including ART adherence, among HIV-infected pregnant women in South Africa. Adherence to antiretroviral medication, which is provided free of charge to all living in South Africa, was defined as taking all ART medication over the last 4 days. This study found that adherence to ART at baseline was associated with higher educational attainment, number of children, time since ART initiation in months, disclosure of HIV status to partner, both psychological and physical partner violence, the use of alcohol, increased stigma, and increased depression. Similar findings are reported in a meta-analysis among women during and after pregnancy [12].

Between the baseline (8–24 weeks pregnant) assessment and the 12 month post-partum assessment, women in the enhanced intervention group underwent two group and three individual cognitive behavioral PYF intervention sessions. The PYF intervention did not have the desired outcome on remaining adherent nor did it have an impact on becoming adherent over time, which was likely due to the *significantly greater proportion* of nonadherent women in the experimental condition at baseline (55 versus 46%). Because this was a cluster-randomized trial, it is likely that clinics randomized to the experimental condition were more likely to have geographic or area-specific factors that may have negatively impacted adherence, with these factors remaining stable by the 12-month follow-up. The study found that there was a change to nonadherence over time. These results, although distressing for the intervention effect, mimic the findings reported in longitudinal studies among HIV infected women in South America [4], Switzerland [5], and in the meta-analysis of 51 articles from across the world [12]. These studies show that self-reported adherence among HIV infected women decreased significantly post-partum.

The reason for the decrease in ART adherence post-partum was postulated in the meta-analysis which found that the mothers concern surrounding the transfer of HIV to her fetus had abated once the baby was born [12]. The necessity-concerns framework [13, 14] clarifies that the mother adhered to her ART regime as she deemed it necessary to protect the fetus from HIV and thus the concerns for the unborn baby far outweighed the concerns about taking the medication. Once the child was born HIV-negative, the mother had no concern for the transfer of HIV from mother to child and therefore may not have been motivated to continue taking her ART medication. At this juncture, the concerns regarding taking ART far outweighed the necessity for taking it. Once this occurred, post-partum ART adherence decreased.

The intervention in this study, as seen in Fig. 1 above, mainly concentrated on adherence during the pre-natal phase and had only one individual session at 6-weeks post-partum. The lack of adherence reinforcement during the post-partum intervention sessions could have negatively impacted the study outcome. At the 6-week post-partum session, high loss to follow up was experienced due to mothers travelling back to their parents’ home for birth. During the study period, the South African government enacted a policy change where Polymerase Chain Reaction (PCR) testing for HIV changed from 6 weeks post-partum to at-birth [37]. This possibly further led to the high loss to follow up that was experienced at 6 weeks post-partum. The high loss to follow up at 6-weeks meant that women did not receive the adherence intervention post-partum.

Although the ART adherence intervention component of PYF did not have the desired overall outcome of increasing adherence over time as mentioned above, it is important to state that the results did show that for those who sustained their ART adherence over time, this was associated with decreased alcohol use, decreased depressive symptoms, and decreased physical intimate partner violence. These associations were also reported elsewhere in literature [12]. In terms of depression, the PYF intervention was effective at reducing depressive symptoms among the study participants [38] which could have led sustained ART adherence. The PYF intervention also had a desired positive outcome on reducing stigma among study participants [39] which is a known determinant of non-adherence.

Reasons given by the most number of women in the enhanced intervention group for missing their ART was that they were away from their home when they needed to take their medication. This is similar to a finding in an Eastern Cape study, which stated that being away from home was their second most important reason for missing ART [11]. In that study population, like this one, when women travelled outside their regular place of treatment, they often travelled without their clinic refill prescription records and thus could not receive ART medication at another clinic. This not only affect those women who travelled in the short term but also those who relocated [11] and as such, women in both the Eastern Cape study and this study ran out of pills. The reason of running out of medication in this study was one of three reasons that increased in proportion from baseline to 12 months post-partum.

The remaining two reasons in the enhanced intervention group that increased in proportion from baseline to 12 months post-partum include having problems taking their medication at specified times and, having problems taking their medication due to the lack of food. These reasons were also stated in the Eastern Cape [11] where the lack of food lead women, who had sufficient ART, to not take their medication. The lack of food worsens ART side effects [11] and thus it would lead women not to adhere to their regimen.

## Limitations of the Study

The study suffered from high loss-to-follow-up due to the migrant population in Mpumalanga. Although the RCT gathered intermediate measures at 32 weeks pre-natal, 6 weeks and 6 months post-natal, the loss to follow-up was too high to warrant analyses of that data for this paper. The number of participants though at baseline and at 12 months post-partum were sufficient for analysis. The measures utilized were subject to self-report recall bias. Some bias was mitigated by utilizing ACASI. The inclusion criteria biased against women without partners as this study was limited only to women who had a partner. The woman’s partner though were not required to be the biological father of the child. Because nonadherence was associated with attrition, it is possible that women who were not adherent may have not been followed up with. Lastly, variables such as knowledge, attitude, norms, skills may have been important to evaluate as these may have influenced adherence.

## Conclusion

The study found that the enhanced intervention had no desired effect on ART adherence over time. It also found that ART adherence decreased post-partum. The high loss to follow up and limited post-partum ART adherence intervention could have led to this outcome. Interventions are thus needed to show the necessity of taking ART post-partum and an increased number of ART adherence interventions are needed during the post-partum phase. Better retention strategies are also necessary at the CHCs as this study was CHC based and structured in such a manner as to only interview women when they came for their regular pre- or post-natal CHC visits. Our high loss-to-follow-up also points to a high loss-to-follow-up of CHC post-partum visits.

Sustained ART adherence was associated with decreased alcohol use, decreased depressive symptoms, and decreased physical intimate partner violence. Due to this finding we recommend that interventions are required to address alcohol use during and after pregnancy, as well as interventions to reduce depression and to increase positive male involvement during and after pregnancy.

## Data Availability

The data for the current study will not be shared publicly as participants were informed at the time of providing consent that only researchers involved in the project would have access to the information they provided.
